# Imaging markers of response to combined BRAF and MEK inhibition in BRAF mutated vemurafenib-sensitive and resistant melanomas

**DOI:** 10.18632/oncotarget.24709

**Published:** 2018-03-30

**Authors:** Stefania Acciardo, Lionel Mignion, Nicolas Joudiou, Caroline Bouzin, Jean-François Baurain, Bernard Gallez, Bénédicte F. Jordan

**Affiliations:** ^1^ Université Catholique de Louvain, Louvain Drug Research Institute, Biomedical Magnetic Resonance Group, Brussels, Belgium; ^2^ Université Catholique de Louvain, Louvain Drug Research Institute, NEST Nuclear and Electron Spin Technologies Platform, Brussels, Belgium; ^3^ Université Catholique de Louvain, Institute de Recherche Expérimentale et Clinique, IREC Imaging Platform, Brussels, Belgium; ^4^ Université Catholique de Louvain, Institute de Recherche Expérimentale et Clinique, Molecular Imaging and Radiation Oncology Group, Brussels, Belgium

**Keywords:** melanoma, tumor response, BRAF/MEK inhibitors, diffusion-weighted MRI, choline spectroscopy

## Abstract

A majority of patients with a V600x melanoma respond quickly to BRAF/MEK inhibition (BRAFi/MEKi) and have an obvious clinical benefit. Nearly all the patients after this initial phase will develop resistance. Therefore, non-invasive early markers of response/non-response are needed in order to identify those patients who, due to intrinsic or acquired resistance, do not respond to treatment and would be eligible for alternative treatments.

The aim of this study was to investigate the value of magnetic resonance spectroscopy (^1^H-MRS) of choline and diffusion-weighted magnetic resonance imaging (DW-MRI) as early markers of response to BRAF inhibition (BRAFi) with vemurafenib alone or in combination with MEK inhibition (MEKi) with trametinib, in BRAFi-sensitive and BRAFi-resistant melanoma xenografts.

Tumor response was significantly improved by the combination of BRAFi and MEKi, compared to BRAFi alone, only in sensitive xenografts; thus indicating that vemurafenib-resistant A375R xenografts were cross-resistant to the inhibition of MEK, as confirmed by immunohistochemistry analysis for phosphorylated ERK.

*In vivo* ^1^H-MRS showed that in sensitive melanoma xenografts, a significant blockage of ERK phosphorylation, but not a decrease in cell proliferation, was required to affect total choline (tCho) levels, thus suggesting that tCho could serve as a pharmacodynamic (PD) marker for agents targeting the MAPK cascade. In addition, early effects of the combination therapy on tumor cellularity could be detected via DW-MRI. In particular, skewness and kurtosis of the apparent diffusion coefficient (ADC) distribution may be useful to detect changes in the diffusional heterogeneity that might not affect the global ADC value.

## INTRODUCTION

Melanoma is the most aggressive skin cancer and has rising worldwide incidence. Although highly curable in its early stages, advanced melanoma has poor prognosis, with a 5 year survival being less than 18% in the presence of metastases [1]. Since its approval in 1975, chemotherapy with dacarbazine has been the standard of care for the majority of patients, despite a limited response rate [2]. The use of chemotherapy has been limited by the introduction of targeted therapies, owing to the discovery of a mutation shared by about 50% of melanomas, consisting in the substitution of valine to glutamic acid in codon 600 in the BRAF gene (activating BRAF^V600E^ mutation) [3]. Besides targeted therapy, immunotherapy has been shown to induce a significant and durable clinical benefit in a limited subset of melanoma patients, providing the rationale for testing BRAF/MEK targeting agents along with immune-checkpoint inhibitors in the clinical settings [4].

Vemurafenib is the first FDA-approved inhibitor of the BRAF mutated serine/threonine kinase [5]. In the BRIM-1, -2 and -3 clinical trials, vemurafenib-treated melanoma patients achieved rapid and unprecedented tumor shrinkage, as well as higher response rates, progression-free survival (PFS) and overall survival compared to dacarbazine chemotherapy [6–8]. Despite the remarkable and rapid tumor regression, responses are in most cases transient, due to development of resistance within 6 to 9 months post treatment initiation [6–8]. Increased MAPK reactivation has been frequently observed in progressing melanomas, providing the rationale for the co-targeting of BRAF and downstream MEK. Indeed, the combinatory regimen prolonged PFS, thus leading to the approval of the combinations vemurafenib/cobimetinib and dabrafenib/trametinib [9]. A third combination involving the BRAF inhibitor (BRAFi) encorafenib and the MEK inhibitor (MEKi) binimetinib has shown in the COLUMBUS phase III trial (NCT01909453) to have the same efficacy than the other two.

Despite the improvements in terms of longer PFS and reduced toxicities, the benefit provided by combined BRAF/MEK targeting is still transient. As aforementioned, most of the mechanisms leading to the onset of resistance rely upon reactivation of the MAPK pathway itself. Paradoxical reactivation of the MAPK pathway may be driven by mutations in NRAS or MEK, amplification or splicing variants of BRAF, elevated levels of CRAF, overexpression of COT. Besides reactivation of the MAPK cascade, resistance to BRAF inhibition can be mediated by activation of the bypass PI3K/AKT/mTOR pathway, mainly via RKT overexpression, PTEN loss or AKT amplification. Notably, tumor microenvironment also plays a role in development of resistance to BRAFi [10]. Moreover, more than 15% of patients do not achieve any benefit at all, due to intrinsic resistance to the treatment.

Given the multiplicity of mechanisms driving resistance, combinational therapies addressing multiple targets within the MAPK cascade, or co-targeting multiple pathways are attractive strategies with an aim to prolong clinical responses. In this context, early non-invasive markers of response are needed in order to identify those patients who, due to intrinsic or acquired resistance, do not respond to treatment and would be eligible for alternative combinations, thus sparing them from ineffective therapies.

The aim of this study was to investigate the value of magnetic resonance spectroscopy (MRS) of choline and diffusion-weighted magnetic resonance imaging (DW-MRI) as early markers of response or resistance to targeted therapies for the treatment of BRAF mutated melanoma. Secondly, we assessed inhibition of the target, cell proliferation and cell death as measured by immunohistochemical staining, and compared them to the magnetic resonance biomarkers.

Alterations in choline metabolism are commonly observed in cancer, compared to healthy tissues. This had initially been regarded as the cellular response to meet the higher demand for membrane turnover, to support increased proliferation rates [11]. Since then, owning to the relationship between proliferation rate and choline levels, MRS of choline has been evaluated as a non-invasive tool for tumor detection/staging in several cancer types, in both preclinical and clinical studies [12].

Besides from its diagnostic potential, total choline may provide insight into response to therapy. In tumor xenograft models, changes in choline levels have been evaluated in response to conventional anticancer treatments [13, 14]. Moreover, by virtue of the relationship between choline metabolism and various oncogenic signalling pathways, choline levels have been exploited as pharmacodynamic biomarkers in preclinical studies, for the monitoring of several targeted therapies [15–19]. Among them, MAPK cascade targeting agents were shown to induce a drop in choline levels, both *in vitro* and *in vivo* [20–23].

In the retrospective study performed by Jagannathan *et al*. on 67 patients, choline signal was present in 78% of cases, and was decreased in 89% of patients undergoing neoadjuvant chemotherapy (NACT), thus providing evidence for its potential as a marker of response of breast tumors to NACT [24]. Subsequently, other studies have confirmed the potential value of choline measurements as a marker of response of breast tumors to chemotherapy [25–27]. In addition to breast tumors undergoing NACT, a drop in tumor choline content has been observed following radiation-induced necrosis in gliomas [28, 29] and MRS of choline has been evaluated in patients with advanced solid tumors, in the Phase I study of the choline kinase alpha inhibitor TCD-717 (NCT01215864).

Diffusion weighted imaging (DW-MRI), based on the Brownian motion of water molecules in both intra- and extra-cellular space, can be used to assess tumor cellularity [30, 31]. Indeed, changes in the apparent diffusion coefficient (ADC) are associated with cell death, and have been observed in a number of different tumors, and treatment modalities, as reviewed in [32, 33]. However, only one preclinical study has evaluated median ADC as a marker of response to the targeting of the MAPK in BRAF mutated tumors [34]. Besides median or mean values, histogram analysis of the ADC distribution may be necessary when dealing with heterogeneous microenvironments [35]. Among the histogram parameters, skewness and kurtosis, which represent the symmetry and the broadness of the ADC distribution, have been associated to response to chemotherapy or anti-angiogenic therapy in patients with ovarian or peritoneal cancer and high-grade gliomas, respectively [36, 37].

In this study we investigated choline MRS and parameters obtained from the histogram analysis of the ADC distribution as early markers of response to MAPK targeting via combined BRAF and MEK inhibition, in BRAF^V600E^ vemurafenib-sensitive and resistant melanoma xenografts.

## RESULTS

### BRAF and MEK inhibition delay the growth of sensitive melanoma xenografts but not of BRAFi-resistant melanoma

**Figure 1:**
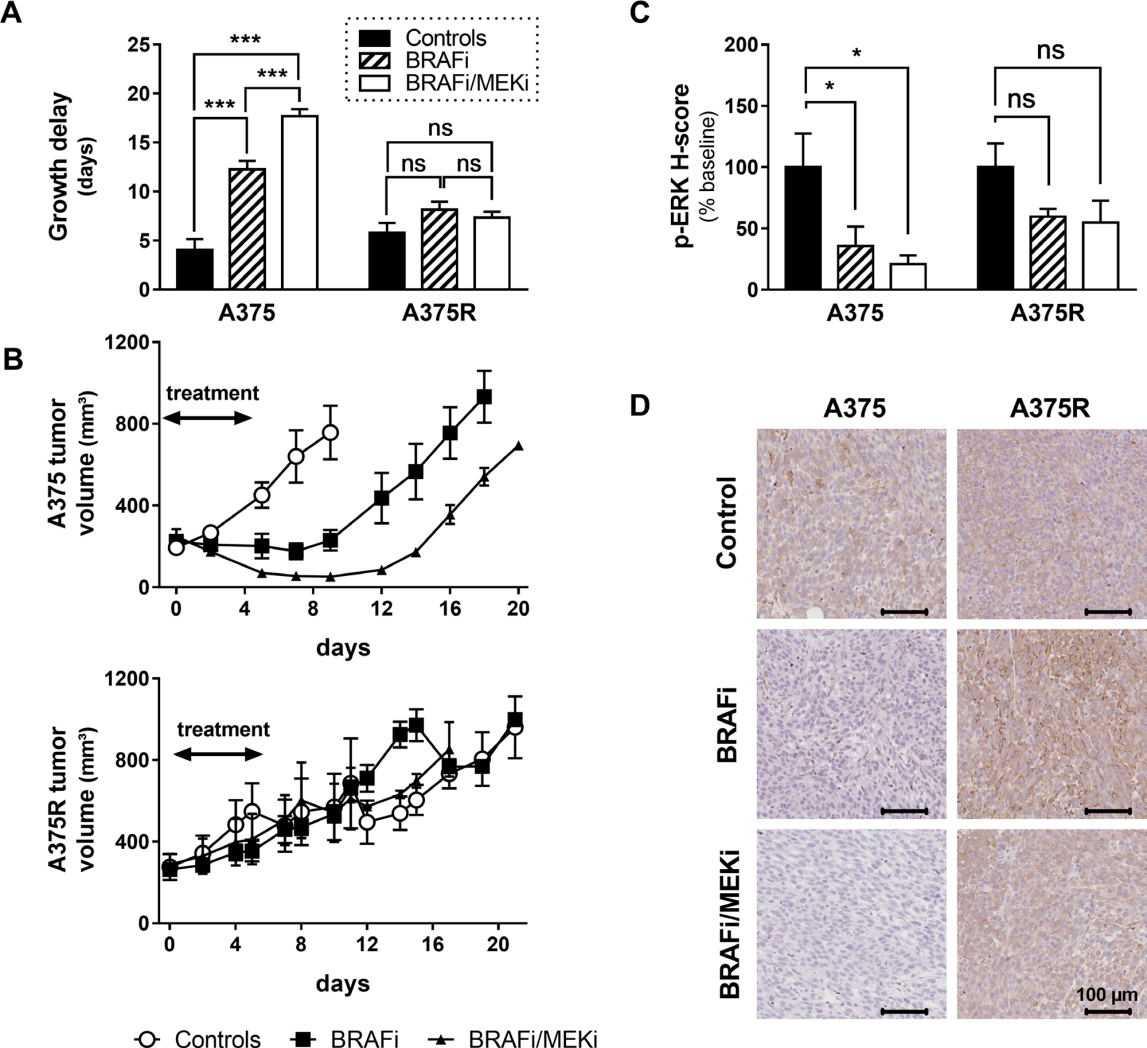
Growth delays and ERK phosphorylation in sensitive and resistant A375 xenografts (**A**) Tumor growth delay was calculated as the mean time for tumors to reach twice their baseline volume. The growth of sensitive A375 xenografts was significantly delayed by both BRAFi as a single agent or in combination with the MEKi (*p <* 0.001, *n* = 3–4/group), with an evident benefit of the combination over BRAFi alone (*p <* 0.001 vs A375-BRAFi). Neither BRAFi alone nor the combination delayed the growth of resistant A375R xenografts (*p* = 0.16 and *p* = 0.65 respectively vs A375R-controls, *n* = 4–5/group). (**B**) Tumor growth curves in BRAFi-sensitive (upper graph) and BRAFi-resistant (lower graph) melanoma xenografts (*n* = 3–5/group). (**C**) Phosphorylation of ERK was significantly blocked in sensitive xenografts collected 4 hours after a single injection of BRAFi alone or in combination with MEKi (^*^p < 0.05 vs A375-controls, *n* = 3/group). As expected, phospho-ERK decrease was not significant in BRAFi-treated A375R xenografts (*p* = 0.2458 vs A375R-controls, *n* = 3). However, the BRAFi-resistant xenografts did not retain sensitivity to the inhibition of MEK either (*p* = 0.1779 vs A375R-controls, *n* = 3). (**D**) Phospho-ERK representative staining of melanoma xenografts collected 4 hours after a single injection of the indicated treatments.

Both BRAFi as a single agent and BRAFi/MEKi combination delayed the growth of sensitive A375 melanoma xenografts, with a significantly longer delay being induced by the combination (4.06 ± 1.89 days in controls, 12.3 ± 1.77 days in BRAFi-treated mice and 17.7 ± 1.43 days in BRAFi/MEKi treated mice). As expected, and contrarily to sensitive xenografts, the growth of A375R xenografts was not significantly delayed by vemurafenib, compared to vehicle-treated A375R xenografts (5.8 ± 2.01 days and 8.15 ± 1.81 days in controls and BRAFi-treated mice, respectively). Notably, the addition of the MEKi did not add any benefit in term of growth delay in BRAFi-resistant melanomas (7.34 ± 1.23 days), thus suggesting that resistance to vemurafenib was accompanied by a cross-resistance to inhibition of MEK (Figure 1A).

Figure 1B shows tumor volume data obtained from sensitive and resistant xenografts treated for 7 days with BRAFi as single agent or in combination with MEKi. To confirm the effective inhibition of the target, we have performed immunohistochemistry for phosphorylated ERK on A375/A375R xenografts collected 4 hours after a single injection of BRAFi alone or in combination with MEKi. Phospho-ERK levels were significantly reduced, by both treatments, only in sensitive xenografts. Coherently with the growth delay results, the BRAFi-resistant A375R xenografts did not retain sensitivity to the inhibition of MEK (Figure 1C). Representative examples for phospho-ERK staining of melanomas collected 4 hours after treatment injection are shown on Figure 1D.

### Combined BRAF/MEK inhibition decreases choline levels in BRAFi-sensitive, but not in BRAFi-resistant, melanoma xenografts

**Figure 2:**
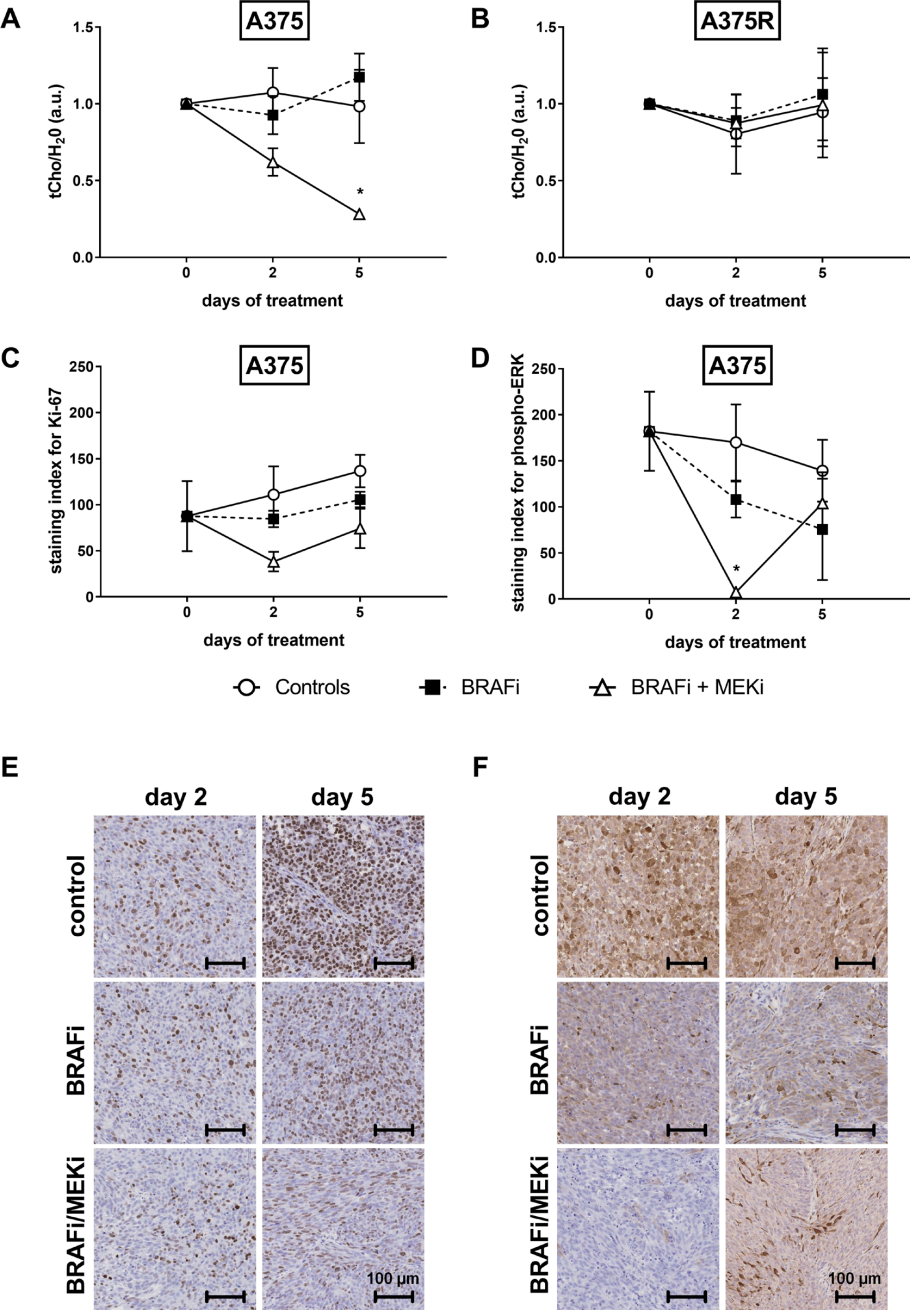
Combined BRAF and MEK inhibition decreases the tCho/H2O ratio in BRAFi-sensitive melanoma xenografts The ratio of total choline peak (tCho) to (unsuppressed) water peak in tumors was measured at baseline, on day 2 and on day 5. Choline levels were affected by the BRAFi/MEKi combination in sensitive (**A**), but not in vemurafenib-resistant (**B**) xenografts (^*^p = 0.0313 vs A375-baseline, *n* = 3–9/group). (**C**) Ki-67 staining was not significantly affected by either treatment (*n* = 3/group, two-way ANOVA followed by Bonferroni's test for multiple comparisons), whereas phospho-ERK levels (**D**) in sensitive A375 xenografts were significantly decreased by the combination as soon as 2 days post treatment initiation (^*^p = 0.0128, *n* = 3–5/group). Representative Ki-67 (**E**) and phospho-ERK (**F**) staining of sensitive melanoma xenografts at baseline and after 2 or 5 days of treatment.

The ratio of total choline peak (tCho) to (unsuppressed) water peak in tumors was measured at baseline, on day 2 and on day 5. Choline levels were affected by the BRAFi/MEKi combination in sensitive (Figure 2A), but not in vemurafenib-resistant xenografts (Figure 2B).

To verify whether the drop in tCho was associated to a decrease in cell proliferation or to the blockade of the MAPK cascade, we performed immunohistochemistry for the proliferation marker Ki-67 and for phosphorylated ERK at the same time points than ^1^H-MRS measurements. Ki-67 levels in control mice slightly tended to increase (127% and 156% of the baseline value after 2 and 5 days), whereas they were stable in the BRAFi treated group. In mice treated with the BRAFi/MEKi combination, the drop in Ki-67 (44% and 85% of baseline at day 2 and 5, respectively) did not reach significance (Figure 2C).

After 2 days of treatment, ERK phosphorylation was completely abrogated in BRAFi/MEKi treated xenografts. Despite the effective inhibition of ERK phosphorylation achieved within 4 hours after a single injection of BRAFi or the combination, phospho-ERK levels were restored at later time points in both cases. Such paradoxical effect was observed after only 2 days of BRAFi treatment, whereas it occurred later in the combination-treated xenografts (Figure 2D).

Representative examples of immunohistochemistry staining for Ki-67 and phospho-ERK in sensitive melanoma xenografts are shown on Figure 2E, 2F, respectively.

According to these findings, a blockade of ERK phosphorylation, rather than a significant reduction in the cell proliferation rate, may be required to affect choline levels.

### BRAFi/MEKi affects diffusional heterogeneity, rather than global diffusion, in sensitive melanoma xenografts

**Figure 3:**
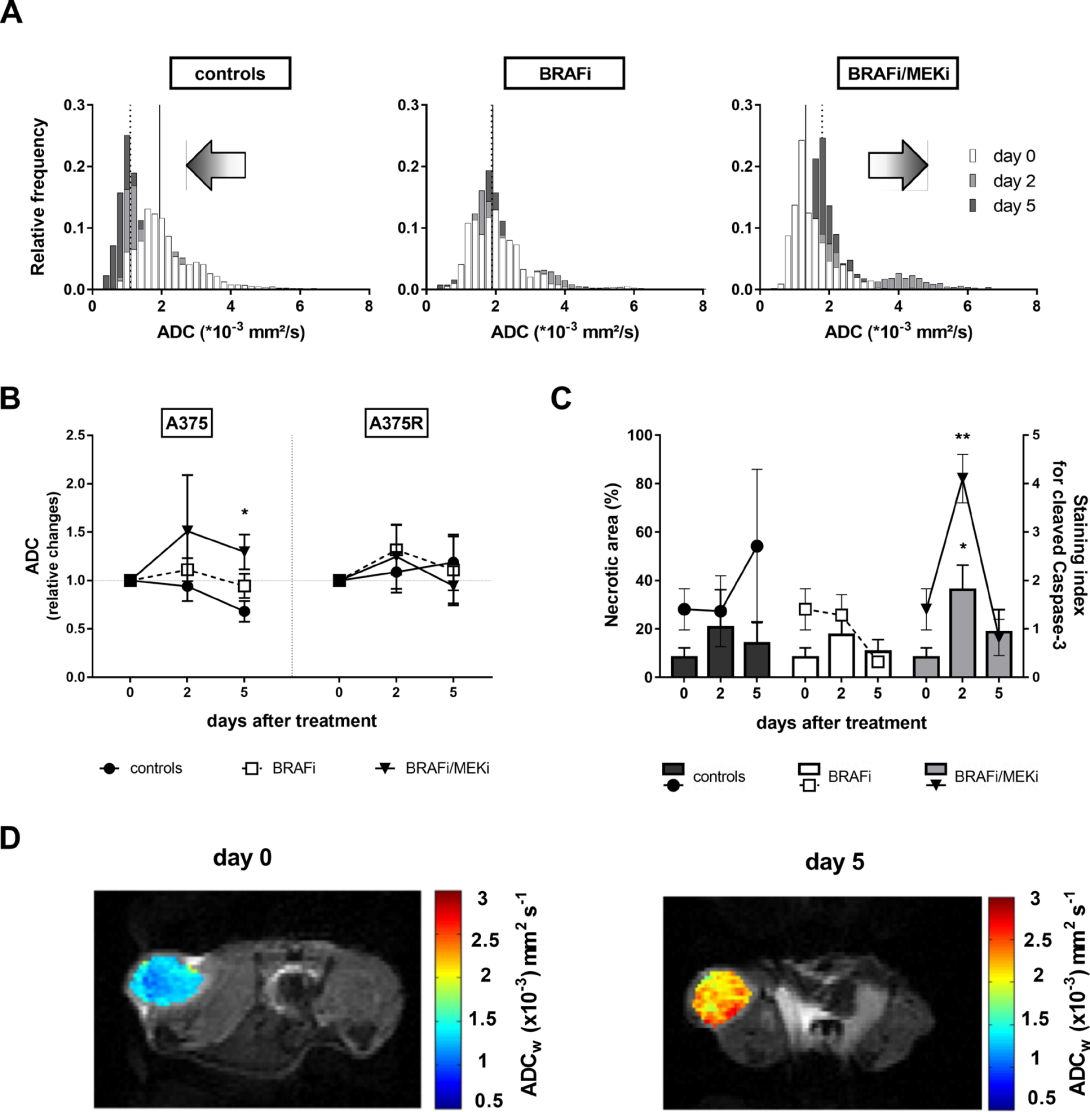
Effects of the BRAFi/MEKi combination on cell death (**A**) Histograms of the ADC values of A375 xenografts obtained from DW-MRI of the same mice as in Figure 4B. The median value for day 0 (full line) and day 5 (dotted line) are shown. Note a clear shift to the left of the median over time in control tumors, a lack of left shift for the BRAFi condition, and a right shift for the combined BRAF and MEK inhibition, as well as a change in the shape of the histograms in treated groups. (**B**) In sensitive A375 xenografts (left), median apparent diffusion coefficient underwent opposite shifts in BRAFi/MEKi-treated A375 xenografts compared to controls (*p* = 0.0517 at day 2, *p* = 0.0333 at day 5 vs A375-controls, *n* = 4–9/group), whereas it was not affected by BRAFi alone. No significant trends were observed in A375R xenografts after treatment (right). (**C**) Necrotic area (bars, left y-axis) in A375 xenografts is represented as percent of total tissue section area (^*^p = 0.0187 day 2 vs baseline, *n* = 3–5/group), whereas cleaved caspase 3 levels (symbols, right y-axis) are represented as mean staining index (^**^p = 0.0082 day 2 vs baseline, *n* = 3–5/group). (**D**) Representative DW images overlaid with corresponding parametric ADC maps acquired at baseline (day 0) and after 5 days of BRAFi/MEKi treatment.

Combined BRAFi/MEKi induced an opposite shift in the apparent diffusion coefficient histograms of BRAFi/MEKi treated A375 mice, compared to controls, thus suggesting a decrease in cellularity in treated xenografts on the one hand, and a simultaneous increase in the cellularity of the untreated group, on the other hand (Figure 3A). This effect led to a significant difference between the corresponding median ADCs after 5 days of treatment (Figure 3B). However, neither in BRAFi- nor in BRAFi/MEKi-treated mice the global ADC value was significantly different from baseline value at day 0.

The trend observed in the global diffusion data was coherent with the *ex vivo* measurements of apoptosis via cleaved Caspase-3 staining and necrosis, since in both cases an increase, even though transitory, was observed following BRAFi/MEKi treatment, but not after BRAFi alone. In vehicle-treated tumors, considerable necrosis was present as well, which was likely due to the rapid tumor growth leading to the formation of a necrotic core (Figure 3C). Representative DW images of a sensitive xenograft at baseline and after 5 days of BRAFi/MEKi treatment are shown on Figure 3D.

We have subsequently investigated whether heterogeneity parameters skewness and kurtosis would have been more sensitive, and therefore suitable for longitudinal monitoring, than global ADC. Skewness represents the symmetry of a distribution. From a physiological point of view, when a tissue image has a positively (towards the right) skewed ADC histogram, most of its pixels correspond to area of low diffusion. This situation could be found in tissues characterized by high cell density, such as tumors. Kurtosis reflects the broadness of a distribution or, said differently, the weight of the tails compared to the central part of the histogram. Homogeneous tissues are more likely to have a narrow ADC distribution, and higher kurtosis values; whereas lower kurtosis may be found in heterogeneous tissues.

**Figure 4:**
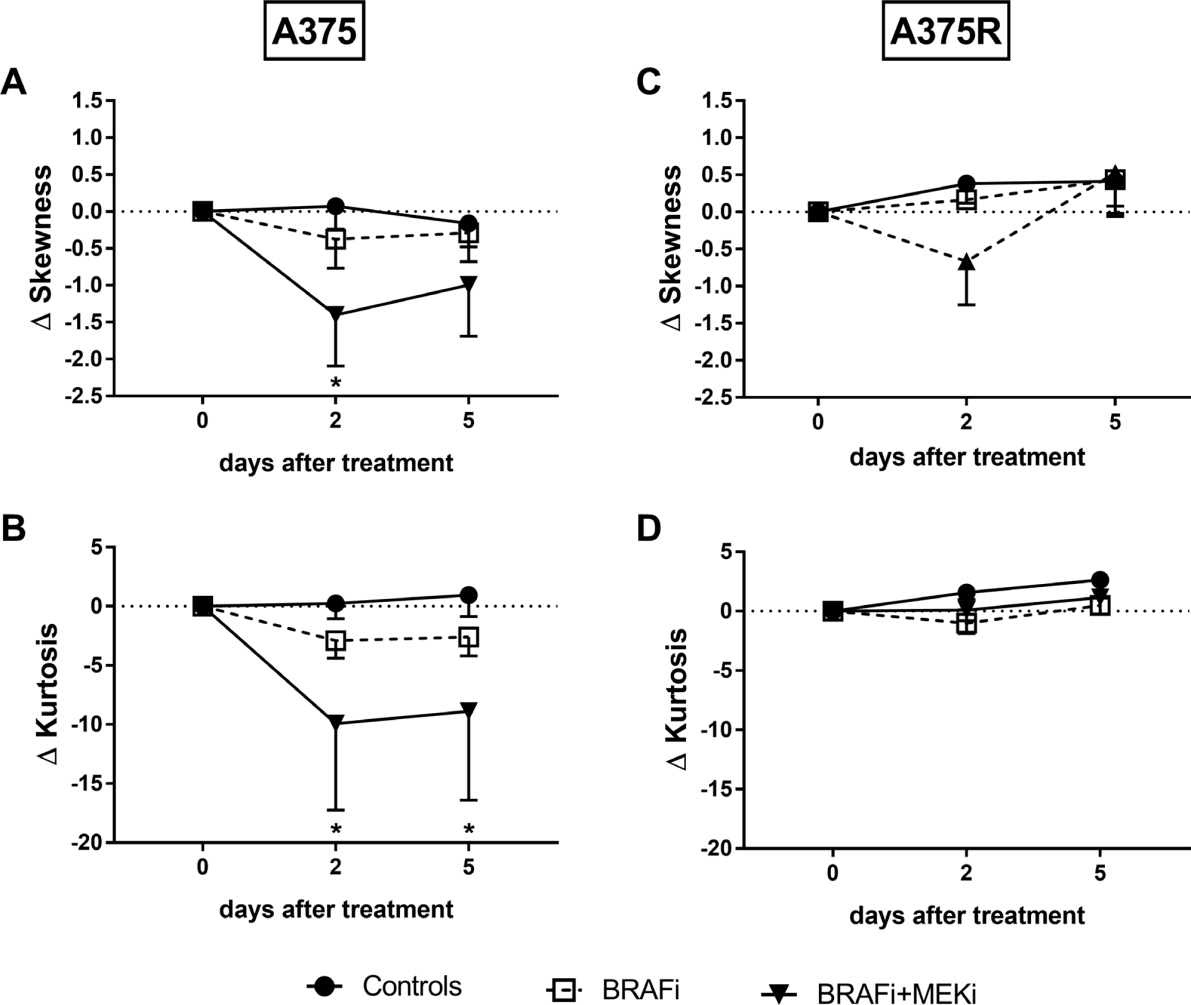
BRAFi/MEKi combination affects diffusional heterogeneity in sensitive melanoma xenografts Skewness (**A**–**C**) and kurtosis (**B**–**D**) of the ADC distribution are represented as absolute differences from baseline (mean ± SEM). Both skewness (^*^p = 0.0210 vs day 0, *n* = 4–9/group) and kurtosis (^*^p = 0.0103 at day 2, *p* = 0.0229 at day 5 vs day 0, *n* = 4–9/group) decreased following combined BRAF/MEK inhibition in BRAFi-sensitive (**A**–**B**), but not in BRAFi-resistant (**C**–**D**) melanoma xenografts (*n* = 4/group).

In sensitive A375 melanomas, both skewness and kurtosis of the ADC histograms were decreased by BRAFi/MEKi combination. In BRAFi-resistant melanomas however, neither BRAF inhibition nor the combination affected skewness or kurtosis (Figure 4). Our results do indicate that these parameters could be used as early markers of response to the simultaneous targeting of BRAF and MEK in melanoma.

## DISCUSSION

The principal aim of the present study was to assess whether MR spectroscopy and diffusion weighted imaging could serve to non-invasively detect response to BRAF/MEK inhibition in BRAF mutated melanoma xenografts. To date, there is a lack of clinically validated markers of response to targeted therapies for melanoma.

With respect to growth delay assays, both BRAFi as a single agent and BRAFi/MEKi combination delayed the growth of sensitive melanoma xenografts, with a significantly longer delay being induced by the combination. The growth of A375R xenografts was non-significantly delayed by vemurafenib, compared to vehicle-treated resistant xenografts. This was reflected by the observed significant decreases in phospho-ERK staining 4 h post-treatment in sensitive xenografts, contrarily to vemurafenib resistant xenografts.

Contrarily to sensitive xenografts, the addition of the MEKi did not add any benefit in term of growth delay in BRAFi-resistant melanomas. Such cross-resistance to MEKi is in agreement with previous *in vitro* studies [38, 39] and it suggests that in our model the BRAFi resistance is neither due to alterations upstream of BRAF nor to alteration in BRAF itself, but it is likely to be due to alteration downstream of BRAF or to bypass signalling pathways which activate ERK, such as the PI3K cascade. In particular, among the numerous and well-described mechanisms of resistance to BRAFi, COT overexpression [40], loss of PTEN [41–43], alterations in the PI3K-AKT pathway, MEK1/2 mutations [44–46] are more likely to explain the concomitant resistance to MEKi observed in our study. Despite being upstream of BRAF, NRAS has also been found to be implicated in the MEKi resistance induced by inhibition of BRAF [39], such apparently controversial observation being explained by the fact that NRAS can signal via both the MAPK and the PI3K cascade.

We have performed IHC to evaluate the relative contribution of cell death and reduced proliferation on the growth delay observed in sensitive xenografts. Vemurafenib has been shown to reduce cell-proliferation as shown by the reduction in Ki-67 levels measured in patient biopsies [47]. In a panel of melanoma cell lines, senescence, but not apoptosis detected via TUNEL assay or cleaved Caspase-3 staining, was the main effect induced by vemurafenib [48]. In addition, MEK inhibition has been suggested to drive apoptosis via a mechanism that does not involve caspases [49]. Cleaved Caspase-3 and Ki-67 analysis revealed a limited contribution of apoptosis in the combination group, and a trend but non-significant effect on proliferation in either BRAFi-treated or combination-treated mice. Even though the exact mechanism, whereby inhibition of BRAF and MEK triggered an arrest in the growth of sensitive xenografts remains to be elucidated, vemurafenib-induced senescence could explain the tumour growth arrest that we observed despite the overall stability of the markers of apoptosis and proliferation. Cellular senescence has been shown to prevent the malignant transformation of BRAF^V600E^ benign naevi [50] and it is implicated in melanoma cell response to BRAF inhibition, as well [48, 51]. Moreover, induction of senescence has been suggested as a therapeutic modality for treatment of vemurafenib-resistant melanomas [52]. Therefore, further experiments to verify the presence of a senescence-associated phenotype in our sensitive and resistant models would undoubtedly contribute to a better understanding of the role of senescence in BRAF^V600E^ melanoma response to treatment.

The change in phospho-ERK levels in tumor samples is currently one of the main outcomes measured in clinical trials testing MAPK targeting agents in melanoma [47, 53]. However, besides being invasive, assessment of phospho-ERK also showed lack of correlation with proliferation in a panel of melanoma cell lines treated with MEK inhibitors [54]. Adaptive sequential approach based on the biopsy of tumors undergoing therapy with BRAF/MEK inhibitors is currently evaluated in clinical studies (NCT02314143).

In the current study, ERK phosphorylation was significantly blocked in sensitive xenografts collected 4 hours after a single injection of BRAFi or the combined BRAF and MEK inhibition. In A375R xenografts, a single injection of the combination of BRAFi/MEKi did not inhibit ERK phosphorylation to a larger extent than BRAFi alone, therefore supporting the hypothesis of a cross-resistance to MEKi in A375R xenografts. Interestingly, phospho-ERK levels in sensitive xenografts were paradoxically restored as soon as 2 days or 5 days post treatment initiation, in BRAFi-treated or combination-treated mice, respectively. This effect occurred while mice were still under treatment and the tumor volume was stable or smaller than at baseline, thus suggesting that the tumor response may not depend exclusively on the abolishment of ERK phosphorylation. This phaenomenon has previously been observed *in vitro*: phospho-ERK levels measured in melanoma cells treated with the BRAFi PLX4720 were completely recovered after 24h of treatment, even though cell growth arrest and apoptotic cell death were still present at later time points, as evidenced by annexin-V flow cytometry, MTT, and cell counting and it was interpreted as the early cell requirement to evade treatment [55]. Few mechanisms of resistance may result in restored phospho-ERK levels following dual BRAF/MEK inhibition as soon as 5 days post treatment initiation: it is unlikely that combination-resistant MEK mutants emerged in such a short time frame. COT can activate ERK both via MEK and independently of MEK, and increased COT levels have been identified as a driver of resistance to BRAFi [40]. Further studies are needed to assess the eventual increase of COT levels in our model, and its correlation with the early restore of ERK phosphorylation.

Taken together, these results suggest that phospho-ERK decrease is required but not sufficient for induction of a tumor response. This provides a valid rationale for the introduction, in the clinical setting, of magnetic resonance markers of response that correlate with response criteria and would provide complementary information to immunohistochemistry, with the additional advantage of allowing longitudinal, non-invasive monitoring.

Within the scope of the present work, total choline levels and diffusion parameters were assessed in response to BRAF/MEK inhibition and our data show a decrease in total choline that becomes significant after 5 days of the combined inhibition of BRAF and MEK.

Since phosphatidylcholine, the major component of plasma membrane, is generated from choline, the higher content of choline in tumors compared to healthy tissues had been initially interpreted as the cancer cell requirement to sustain its increased proliferation rate. In our study, a significant inhibition of ERK phosphorylation, but not an effect on the proliferation marker Ki-67 or a significantly longer tumor volume doubling time, was required to affect tCho. These findings suggest that tCho variation observed in our model was not driven by a decrease in the proliferation rate, and that tCho is rather a PD marker for the targeting of the MAPK pathway.

This is in line with the fact that the Kennedy pathway, which is behind choline synthesis, interacts with various oncogenic signaling pathways, such as the MAPK and the PI3K cascades, thus allowing to exploit choline also as PD marker, to follow the action of molecularly targeted agents, which may precede the effect on proliferation. The decrease in choline levels has already been exploited as PD markers for several inhibitors of PI3K/mTOR [15, 56–60], MEK [20, 21], HIF-1α [18] as well as for silencing, or pharmacological inhibition, of choline kinase [19, 61, 62].

In a recent study, and contrarily to our findings, neither ^31^P NMR spectra nor ^1^H MRS showed any effect of BRAFi on either choline, phosphocholine or glycerolphosphocholine in WM266.4 cells, despite the effect on ERK phosphorylation [63]. Although not performed on the same cell line and not assessed at several time points, the reason behind the stability of choline levels in the work of Delgado-Goni *et al*. could be due to the possibility that choline drop is not induced by the targeting of the MAPK, ultimately resulting in the abolishment of ERK phosphorylation, but it is specific to the inhibition MEK. Therefore, despite the evidence of a close relationship between the MAPK cascade and choline metabolism, which allows to exploit choline-containing compound as non-invasive PD markers for the inhibition of MEK, the exact mechanism by which those agents affect choline metabolism remains to be elucidated. Because of the indirect and complex interplay between the Kennedy pathway and the MAPK signalling cascade, it is difficult to discriminate the relative contributions of all the mechanisms leading to the decrease in tCho.

In the clinical settings, although 1H-MRS is routinely performed along with MRI in patients with brain, breast and prostate tumors [64], the introduction of choline levels as a biomarker is limited by the difficulty to obtain good SNR in small lesions[64–66, 27], presence of tumor size effect [67], and lack of standardization among protocols and thresholds used by different centers [68, 69].

Besides choline spectroscopy, apparent diffusion coefficient (ADC) calculated using diffusion-weighted imaging can be exploited as marker of response to anticancer treatment, owing to the correlation between cellularity and diffusion of water molecules within a tissue. In sensitive A375 xenografts treated with the combination, median ADC was significantly increased over time, compared to the vehicle treated counterpart. However, such increase was not significant compared to the baseline value, indicating that the observed effect was rather due to a simultaneous increase in diffusivity in the treated mice and a decrease in controls, where diffusion may be hindered by hypercellularity. This result was consistent with IHC data of cleaved caspase 3, and it confirms the value of DW-MRI as a tool for the detection of cell death; however, the technique may not be powerful enough for a longitudinal monitoring of melanoma response to BRAF/MEK inhibition in a clinical context.

Besides mean/median ADC, a more thorough analysis of the assessment of histogram parameters including first-order statistics, such as standard deviation, coefficient of variation and quantiles, as well as higher order statistics, such as skewness and kurtosis, may improve the assessment of response to treatment [35, 70]. In our study, both skewness and kurtosis of the ADC distribution decreased following combined BRAF and MEK inhibition in sensitive, but not in resistant, xenografts. These results are in keeping with other findings on ADC histogram analysis, and they highlight the importance of evaluating diffusivity in a comprehensive way, by looking not only at changes in the global ADC, but also at the histogram parameters depicting the diffusional heterogeneity.

Elevated baseline values of skewness and kurtosis of the ADC distributions were predictive of local failure in patients with head and neck carcinoma undergoing radiation therapy alone or combined with chemotherapy [71]. Not only skewness and kurtosis have been suggested as predictive/prognostic markers, but also as marker of response to treatment. An increase in ADC, followed by a decrease in skewness and kurtosis, has been observed in the ADC histograms of metastatic ovarian and primary peritoneal cancer patients who responded to chemotherapy [36]. In another retrospective study, increased or stable/decreased skewness after bevacizumab/irinotecan therapy was found in responding and in progressing patients, respectively [37]. More recently, a decrease in skewness and kurtosis, although the latter did not reach significance, has been observed in cervical cancer patients undergoing radiation therapy (NCT01992861) [72]. Another study on cervical cancer patients is currently aiming at evaluating several histogram parameters, including skewness and kurtosis, to assess their potential as prognostic biomarkers (NCT01937533).

In regard to DW-MRI, our work highlights the usefulness of accompanying ADC measurements with histogram analysis of ADC distribution for longitudinal monitoring, and the potential value of such histogram markers for the monitoring of melanoma patients treated with MAPK targeting agents. ADC as response biomarker is increasingly being integrated into clinical trials, in particular in breast cancer patients undergoing neoadjuvant treatments (NCT02798484, NCT01564368, NCT02916719). Compared to breast, the acquisition might be more challenging in case of melanomas in the abdomen region, due to respiration and motion artefacts.

## CONCLUSIONS

Our study strengthens the fact that the decrease in phospho-ERK levels is not likely to be an optimal marker of response to BRAF and MEK inhibition in melanoma as such condition seems necessary but not sufficient to achieve response to treatment and as phospho-ERK levels may be paradoxically restored during treatment. In addition, in our study, neither Ki-67 nor cleaved caspase 3 immunohistochemistry markers underwent significant variations in response to treatment, despite the presence of a considerable tumor growth delay. Besides the fact that immunohistochemistry provides information about a limited section of the tumor, whereas MR-based markers take into account the whole tissue, an hypothesis is that cellular senescence induced by MAPK inhibitors could play a role on the observed growth delay, although this would need to be further investigated.

In the light of our findings, we suggest that ^1^H-MRS of choline can be exploited as a pharmacodynamic marker for the targeting of the MAPK cascade by BRAF inhibitors combined with MEK inhibitors whereas changes in diffusional heterogeneity induced by the combination could help to discriminate between sensitive and resistant tumors. Further studies confirming the value of skewness and kurtosis of the ADC distribution as markers of response to BRAFi/MEKi, will help to optimize the early detection of therapeutic response, as well the design of novel combination strategies.

## MATERIALS AND METHODS

### Tumor models

A375 human malignant melanoma cell line was purchased from Sigma-Aldrich (USA) and cultured in Dulbecco's Modified Eagle Medium (DMEM) containing high glucose, HEPES, and supplemented with 10% heat-inactivated fetal bovine serum (Thermo Fisher Scientific, USA). The BRAFi-resistant cell line (A375R) was generated *in vitro* by exposing the parental A375 cell line to increasing concentration of PLX-4032 (vemurafenib) for 14 passages, starting from 1 μM and up to 7.5 μM. A375R cells were kept in culture in 2.5 μM PLX-4032.

Cells were harvested by trypsinization, counted with the trypan blue exclusion method, and resuspended in PBS (pH 7.4) before injection in animals. 2.10^6^ A375 or A375R cells in 100 μL of PBS were subcutaneously injected in the right hind paw of 6-week old female nude NMRI mice (Janvier, Le Genest Saint Isle, France).

### Animal treatment

Following tumor inoculation, when the shortest xenograft diameter reached 4 mm, mice were randomized into 3 groups and treated via daily intraperitoneal injections of the BRAF inhibitor PLX-4032 (50 mg/kg) or the combination of PLX-4032 and the MEK inhibitor GSK-112021 (0.5 mg/kg) or vehicle (35 μL DMSO/100 μL PBS). PLX-4032 (vemurafenib) and GSK-112021 (trametinib) were purchased from Active Biochem (Hong Kong). After 7 days, treatments were interrupted, and tumor regrowth was longitudinally monitored using calipers. The growth delay of melanoma xenografts was calculated as the time, in days, to reach twice the baseline volume. During MR experiments, animals were kept under inhalational anesthesia with isoflurane (2.5% during anesthesia induction, 1–2% during maintenance) in 2 L/min airflow. Temperature was continuously monitored and kept at 37°C ± 1°C via a warmed blanket.

Experiments involving animals were undertaken in accordance with the Belgian law concerning the protection and welfare of the animals and were approved by the Université catholique de Louvain ethical committee (agreement reference: UCL/2014/MD/026). All investigators performing *in vivo* studies successfully completed FELASA C training.

### ^1^H-MRS of choline

*In vivo* ^1^H-MRS was performed at baseline, and after 2 and 5 days of treatment on an 11.7 tesla MR system (Bruker, Ettlingen, Germany). Choice of voxel geometry was based upon T2-weighted anatomical images (slice thickness = 1 mm, FOV: 30 mm × 30 mm, matrix size = 256 × 256, TE/TR = 30 ms/2500 ms, RARE factor = 8, NA = 2). Following localized shimming, point-resolved spectroscopic measurement (PRESS) were performed prior and post water suppression (cubic voxel with 3 to 4 mm side, TE/TR = 16 ms/2500 ms, 256 averages, 2048 points). Preprocessing and quantitation of *in vivo* ^1^H-MRS data were performed using the jMRUI software package: unsuppressed water peaks were quantified via the AMARES (Advanced Method for Accurate, Robust and Efficient Spectral) fitting, whereas choline peaks were quantified via QUEST (quantitation based on QUantum ESTimation). Prior QUEST, residual water signal was removed via Hankel Lanczos Squares Singular Values Decomposition (HLSVD) filtering.

### DW-MRI

Diffusion-weighted MR images were acquired using an echo-planar imaging sequence with 6 b-values (100, 200, 400, 600, 800 and 1000 s/mm^2^) and diffusion gradients applied in the x, y, and z directions. Slices were positioned based upon T2-weighted anatomical images, in order to cover the whole tumor. Acquisition parameters were: slice thickness = 1 mm, FOV = 30 mm × 30 mm, matrix size = 128 × 64, TE/TR = 27 ms/3000 ms, NA = 1. ADC maps and median apparent diffusion coefficients were obtained from ROIs covering the whole tumor sections, using a homebuilt Matlab routine (The MathWorks Inc., USA), as previously described [23]. Histogram analysis of the ADC distributions was performed in GraphPad Prism 7 (GraphPad Software, USA).

### IHC

Melanoma xenografts were fixed in 4% formaldehyde for 24 hours and embedded in paraffin. Following deparaffinization, inactivation of endogenous peroxidases, antigen retrieval in citrate buffer and aspecific binding blocking, 5 μm sections were incubated overnight at 4°C with the primary antibodies for phospho-ERK (Cell Signaling Technology, ref. #4370, 1:200 dilution), Ki-67 (Cell Signaling Technology, ref. #9027, 1:600 dilution) or Cleaved Caspase 3 (Cell Signaling Technology, ref. #9661, 1:300 dilution). Consequently, sections were incubated at room temperature for 30 minutes with Envision anti-rabbit secondary antibody (Dako, ref. #K4003) and stained with diaminobenzidine for 5 min (Dako, ref. #K3468). Stained slides were then digitalized using a SCN400 slide scanner (Leica Biosystems, Wetzlar, Germany) at X20 magnification and analyzed using TissueIA (Leica Biosystems, Dublin, Ireland). The quantification algorithm was run in the viable part of the tissue samples to detect stained area and tissue area. A staining index was calculated as the average staining intensity of the positive pixels multiplied by the positively stained area fraction. Regions of interest corresponding to necrotic areas were manually defined, and for each slide necrotic fraction was calculated as the sum of all necrotic areas divided by the total tissue area.

### Statistical analysis

Unless otherwise specified, graphs are plotted as mean with error bars denoting standard error of mean (SEM). Two-way analysis of variance ANOVA, followed by Bonferroni's test for multiple comparisons, was performed in GraphPad Prism 7, with *p* ≤ 0.05 considered significant.
